# From Cell Interactions to Bedside Practice: Complete Blood Count-Derived Biomarkers with Diagnostic and Prognostic Potential in Venous Thromboembolism

**DOI:** 10.3390/jcm14010205

**Published:** 2025-01-02

**Authors:** Emma Eugenia Murariu-Gligor, Simona Mureșan, Ovidiu Simion Cotoi

**Affiliations:** 1 Doctoral School of Medicine and Pharmacy, George Emil Palade University of Medicine, Pharmacy, Science, and Technology of Targu Mures, 540142 Targu Mures, Romania; emma-eugenia.murariu@umfst.ro; 2 Internal Medicine IV, George Emil Palade University of Medicine, Pharmacy, Science, and Technology of Targu Mures, 540142 Targu Mures, Romania; 3 Department of Pathophysiology, George Emil Palade University of Medicine, Pharmacy, Science, and Technology of Targu Mures, 540142 Targu Mures, Romania; ovidiu.cotoi@umfst.ro; 4 Department of Pathology, Mures County Clinical Hospital, 540011 Targu Mures, Romania

**Keywords:** venous thromboembolism, deep vein thrombosis, pulmonary embolism, neutrophil-to-lymphocyte ratio, platelet-to-lymphocyte ratio, systemic immune-inflammation index

## Abstract

Venous thromboembolism (VTE), encompassing deep vein thrombosis and pulmonary embolism, is a significant burden on health and economic systems worldwide. Improved VTE management calls for the integration of biomarkers into diagnostic algorithms and scoring systems for risk assessment, possible complications, and mortality. This literature review discusses novel biomarkers with potential diagnostic and prognostic value in personalized VTE management. The pathophysiology of thrombosis starts with cell interactions in the vascular environment and continues with more complex, recently discussed processes such as immunothrombosis and thromboinflammation. Their clinical applicability is in the use of complete blood count (CBC)-derived immuno-inflammatory indices as attractive, readily available biomarkers that reflect pro-thrombotic states. Indices such as the neutrophil-to-lymphocyte ratio (NLR = neutrophil count divided by lymphocyte count), platelet-to-lymphocyte ratio (PLR = platelet count divided by lymphocyte count), and systemic immune-inflammation index (SII = NLR multiplied by platelet count) have demonstrated predictive value for thromboembolic events. Nevertheless, confounding data regarding cutoffs that may be implemented in clinical practice limit their applicability. This literature review aims to investigate neutrophil and platelet interactions as key drivers of immunothrombosis and thromboinflammation while summarizing the relevant research on the corresponding CBC-derived biomarkers, as well as their potential utility in day-to-day clinical practice.

## 1. Introduction

Venous thromboembolism (VTE), clinically presenting as deep vein thrombosis (DVT) and/or pulmonary embolism (PE), is a significant burden on health and economic systems worldwide, as its incidence increases with age [1,2]. The management of venous thromboembolism in an aging population in a cost-efficient manner requires adequate risk stratification for timely diagnosis in order to provide the appropriate treatment and minimize the risk of long-term complications (e.g., post-thrombotic syndrome after DVT or chronic thromboembolic pulmonary hypertension after PE). The use of pretest clinical probability scores (e.g., the Wells DVT score [1], Wells rule for PE [2], and revised Geneva rule for PE [2]) or rule-out tools (e.g., Pulmonary Embolism Rule-out Criteria (PERC) [2]) can orient the diagnostic algorithm while limiting unnecessary tests. Diagnosis confirmation is followed by the choice of appropriate treatment in terms of the regimen, dose, and duration according to the predisposing risk factors [1,2]. A VTE severity assessment is based on clinical scores. In pulmonary embolism, the most frequently used validated clinical scores are the Pulmonary Embolism Severity Index (PESI) and its simplified version (sPESI), which offer reliable identification of patients at low risk of 30-day mortality as their main advantage [2]. Nevertheless, as the European Society of Cardiology (ESC)’s 2019 Pulmonary Embolism Guidelines [2] emphasize, there is an evidence gap when discussing PE’s severity and risk of early death, as “the optimal, clinically most relevant combination (and cutoff levels) of clinical and biochemical predictors of early PE-related death remain to be determined”. Therefore, VTE is more than a cardiovascular syndrome, and it “may be viewed as part of the cardiovascular disease continuum” [2].

As risk factors for venous thromboembolism—such as platelet count, anemia, and infection—have previously been identified [1], the current study aims to determine whether complete blood count (CBC) parameters and CBC-derived immuno-inflammatory indices can be used alone or integrated into clinical prediction scores to guide clinical reasoning, as well as to assess the burden of venous thromboembolism in terms of morbidity and mortality. The availability and low cost of complete blood count monitoring would allow for readily calculating indices such as the neutrophil-to-lymphocyte ratio (NLR = the neutrophil count divided by the lymphocyte count), systemic immune-inflammation index (SII = the NLR multiplied by the platelet count), and platelet-to-lymphocyte ratio (PLR = the platelet count divided by the lymphocyte count), with potential benefits for the clinical management of venous thromboembolism cases.

## 2. From Cell Interactions in the Vascular Environment to Clinical Practice

### 2.1. Neutrophils and Platelets at the Crossroads Between Immunity and Inflammation Versus Hemostasis and Thrombosis

When discussing cell lines involved in immunity and coagulation leading to the generation of thrombi, the following two emergent terms are sometimes interchanged: immunothrombosis and thromboinflammation. However, as Schrottmaier et al. [3] clarified in a 2024 review, “immunothrombosis refers to the influence of the immune system on the formation of a thrombus, whereas thromboinflammation refers to the impact of the thrombus on the immune system” [3].

The term “immunothrombosis” was first proposed by Engelmann and Massberg in 2013 [4]. According to the abovementioned authors, immunothrombosis can be defined as a “physiological type of thrombosis” that aims to “support innate immune defense”, during which immune cells with procoagulant activity, as well as thrombosis-specific molecular mediators, play a pivotal role. The ultimate goal is to create an intravascular scaffold (composed of fibrin and blood cells) that limits pathological processes (triggered either by pathogens or altered self-components) to the intravascular compartment. However, dysregulated immunothrombosis may contribute to pathological thrombosis [4].

The main cellular components of immunothrombosis are polymorphonuclear cells, platelets, and monocytes, as they express pattern recognition receptors (PRRs) that recognize “non-self” components, based on pathogen-associated molecular patterns (PAMPs) and damage-associated molecular patterns (DAMPs) from damaged host cells [5]. Neutrophils and platelets both act as mediators of thromboinflammation, and the formation of platelet–neutrophil aggregates (PNAs) promotes obstruction and inflammation at the microvascular level [6]. Zhou et al. [7] identified significantly higher amounts of platelet–neutrophil aggregates as well as platelet activation levels in deep vein thrombosis patients compared to a non-DVT group, with PNA levels correlating with the level of total platelet activation. Platelet–neutrophil aggregates were also shown to predict the potential risk of DVT occurrence when a PNA cutoff level of >7.4% was employed (odds ratio = 3.60) [7].

**Figure 1:**
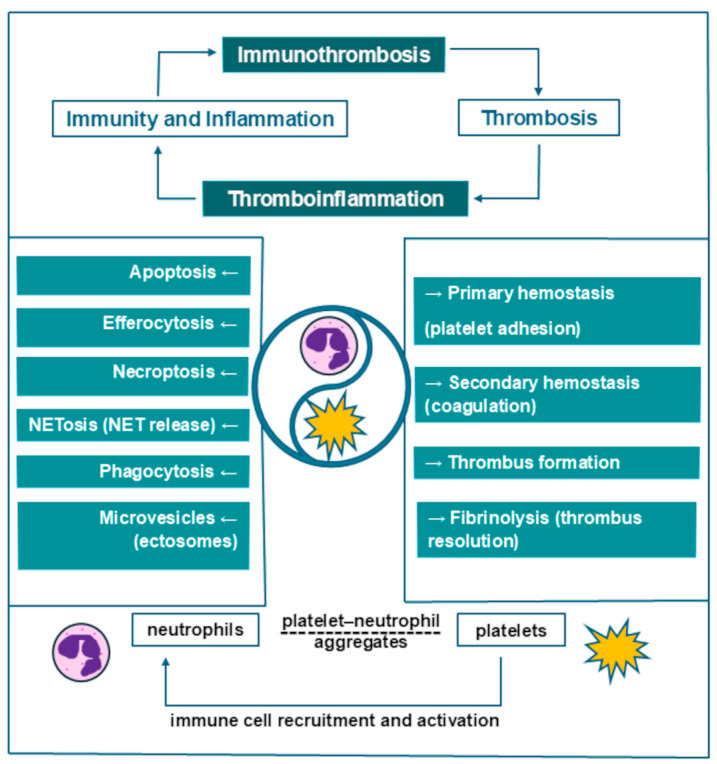
Neutrophils and platelets as key drivers of thrombosis.

Neutrophils’ behavior in the inflammatory environment reflects their phenotypic and functional heterogeneity. According to a 2024 review by Rizo-Téllez et al. [8], neutrophils can act as “friend or foe”, depending on context; this led to the formulation of the ”neutrophil paradox”, describing the dichotomy of their defense versus damage-inducing abilities. Fine mechanisms regulate neutrophils’ activity from their recruitment at the affected site and activation in the initial phase of inflammation to the repair phase. Neutrophils undergo processes such as phagocytosis; NETosis, based on the release of neutrophil extracellular traps (NETs); apoptosis; efferocytosis, based on macrophages removing apoptotic neutrophils in order to end the inflammatory reaction; necroptosis, or programmed necrosis; and the release of microvesicles, also known as ectosomes, which promote communication with the surrounding cells [8] (Figure 1).

Platelets have been described as “the sentinels of vascular integrity” [5]. Platelets promote the recruitment of other immune cells and assist in the formation of intravascular thrombi, together with monocytes and neutrophils [5]. In addition to the well-known role of platelets in primary hemostasis via platelet adhesion, they also intervene in secondary hemostasis (coagulation), fibrinolysis, and immunothrombosis (Figure 1). Upon activation, platelets mediate venous thrombosis and thrombus resolution, as well as vessel wall remodeling [9]. In addition, platelets are the main source of procoagulant microparticles (MPs) (70–90% of all circulating MPs) [9]. In this regard, Vazquez-Garza et al. [5] described a cell surface-based model consisting of initiation, amplification, and propagation phases, thereby reshaping the traditional coagulation model of the tissue factor (extrinsic) pathway and contact activation (intrinsic) pathway. The process is described as starting on tissue factor-exposing cells and on the surface of activated platelets; it is then followed by the interaction of tissue factor with coagulation factors, followed by further platelet activation and aggregation during amplification, as well as consequent propagation mediated by participating coagulation factors [5].

In addition to circulating cells, during venous thrombosis, an important role is played by the endothelial tissue. Inflammation induces endothelial cell activation, with consequent activation of platelets and a clotting cascade [5]. Assessment of endothelial dysfunction in this context can be performed non-invasively using the flow-mediated dilation technique [10]. This method is based on a nitric oxide-mediated dilation of the arterial bed as a reflection of the endothelium’s reaction to a blood flow increase and augmented shear stress. For increased measurement reproducibility, consensus guidelines regarding appropriate assessment techniques were formulated in 2019 [10]. Previous research has emphasized the importance of endothelial dysfunction in immunothrombosis and thromboinflammation. Significantly higher flow-mediated dilation values for the brachial artery were observed in a study published by Kurtipek et al. [11], investigating patients with acute PE compared to healthy controls. Their study also observed significantly higher NLR and PLR values in the pulmonary embolism group [11].

### 2.2. Quest for Accessible and Reliable Biomarkers—Reshaping the Importance of Routine Complete Blood Counts

Risk factors for venous thromboembolism have been widely discussed and researched, but the question of variable susceptibility in certain populations is still debated.

In a 2024 Mendelian randomization study of European patients, Jiang et al. [12] investigated susceptibility to acute pulmonary embolism, identifying genetic associations between circulating blood cell counts and lymphocyte subsets. Causal associations with PE susceptibility were described for decreased levels of circulating white blood cells (OR = 0.88), low lymphocyte levels—particularly low HLA-DR+ natural killer cells (OR = 0.9)—and low neutrophils (OR = 0.88) [12]. Nevertheless, as genetic testing is not widely available, in recent years, many studies have oriented towards more cost-efficient and readily available risk assessment tools based on affordable biomarkers.

The definition of a “biomarker” (or biological marker) varies according to the specific context. Broadly speaking, the term refers to a defined, measurable characteristic that is indicative of a process or a response to exposure/intervention and is different from a clinical outcome assessment. Biomarker subtypes include diagnostic, monitoring, and pharmacodynamic/response biomarkers; predictive, prognostic biomarkers; and safety, susceptibility/risk biomarkers [13].

Complete blood count-derived immuno-inflammatory indices are easily attainable, versatile biomarkers that have been extensively studied in a wide range of pathologies over the past few years. Defined either as ratios between different absolute cell numbers (e.g., neutrophil-to-lymphocyte ratio, platelet-to-lymphocyte ratio) or as compounded indices (e.g., the systemic immune-inflammation index—defined as the NLR multiplied by the platelet count), these indices are still subject to controversy, as different studies have reported various cutoffs for diagnostic and prognostic purposes.

The rationale for their use is based on the interaction of immune system components (innate neutrophil granulocytes versus adaptive lymphocytes) with the vegetative nervous system (sympathetic and parasympathetic) and the neuroendocrine system (stress hormones) in response to various insults. Most often, this results in neutrophilia and lymphocytopenia, leading to a mathematical increase in NLR and, consequently, SII [14].

The current challenge of defining normal range intervals for the abovementioned indices is the need to consider baseline variations according to gender, age, race, stress, and comorbidities, as well as the studied outcomes that justify the differences in reported cutoffs [14]. Mean reference baseline NLR, PLR, and SII values reported across several studies are presented in Table 1.

A 2021 review on exercise physiology by Walzik et al. [18] showed that NLR, PLR, and SII values vary with exercise as a consequence of exercise-induced neutrophilia, lymphocytopenia, and thrombocytosis. NLR, PLR, and SII tended to increase after acute exertion, but NLR and SII exhibited decreased baseline values after 3–4 weeks of chronic exercise, suggesting an anti-inflammatory effect of regular physical training [18].

Another extensive review from 2021 by Zahorec et al. [14] proposes an ”NLR meter”—a scale to quantify NLR values, reflecting the intensity of immuno-inflammatory response in various pathological conditions, including stress, injury, major surgery, trauma, infection, and systemic inflammation, with proposed normal NLR values between 1 and 2, while pathological values were considered to be above 3.0 or below 0.7 in adults. As NLR has shown high sensitivity but low specificity, the authors recommended that NLR cutoff values be refined for each diagnosis. More than its baseline value, the quick variation in NLR in response to insults, along with its association with the clinical evolution in a wide range of inflammatory states, suggests its potential use as a dynamic parameter, with the focus redirected towards variations in NLR rather than its absolute values, irrespective of the specific pathology involved [14].

#### 2.2.1. Biomarkers with Proposed Predictive Value for Venous Thromboembolism

Adequate thromboprophylaxis in populations at risk of a venous thromboembolic event requires correct risk stratification. Risk assessment models (RAMs) have been proposed in this regard, according to the population’s classification as medical (non-surgical), surgical, or ambulatory cancer patients [19,20]. These scoring systems help individualize prophylaxis for venous thromboembolism by considering risk factors, comorbidities, and biomarkers with validated cutoffs. For instance, medical patients benefit from scores such as the Padua Prediction Score [21], the International Medical Prevention Registry on Venous Thromboembolism score (IMPROVE VTE) [22], and the IMPROVE-DD score [23]. In the case of the latter, the quantitative measurement of D-dimer is used for patients’ stratification, with a cutoff established at two times the superior limit of normal [23]. In surgical patients, the Caprini score [24] is the recommended scoring system, while in cancer, the most widely used is the Khorana score [25], which is applicable to ambulatory cancer patients [19,20]. Other RAMs proposed for cancer patients include the Vienna-CATS nomogram score [26] and the COMPASS-CAT score [27].

Cancer is a well-recognized risk factor for thrombotic events [1,2,28]. The venous thromboembolic risk in patients with cancer is nine times higher than that in the general population [28]. The risk of developing cancer-associated thrombosis varies with the type of cancer, and it increases in association with cardiovascular disease or cardiovascular risk factors or when surgical procedures are required. Patient stratification using risk assessment models permits the identification of cases that would benefit from thromboprophylaxis, such as high-risk populations. Risk assessment models are based on various clinical predictors and +/− biomarkers [28]. Validated RAMs that include complete blood count parameters are presented in Table 2.

From the pathophysiological point of view, cancer cells induce a hypercoagulable state through the expression and release of procoagulant molecules, as well as the activation of platelets, leukocytes, and endothelial cells [28]. Cancer patients commonly present an inflammatory status that prompts modifications of the adaptive immune system, reflected in the possible occurrence of neutrophilia and relative lymphocytopenia, with a worse overall cancer prognosis [29]. However, neutrophils infiltrating solid tumors belong to different subsets—either anti-tumorigenic (N1) or pro-tumorigenic (N2)—as part of the tumor-associated neutrophil population [8]. Consequently, a simplistic approach to the predictive value of absolute neutrophil numbers in oncological pathologies is insufficient, and assessment of interferences with other cell lines is of utmost importance. Even so, there is no “one size fits all” approach; the thrombotic risk in oncological patients varies with the tumor type, as also reflected in the variation in the cutoffs of proposed CBC-derived indices.

For oncological patients who do develop a VTE event, mortality rates increase two- to threefold. According to the 2023 European Society for Medical Oncology (ESMO)’s Clinical Practice Guidelines regarding venous thromboembolism in cancer patients [28], it is recommended to use diagnostic imaging methods (compression ultrasonography for DVT or computed tomography pulmonary angiography for PE) whenever there is a suspicion of VTE (class I level of evidence, grade A recommendation), as “in cancer patients the performance of clinical decision rules and D-dimer testing is poor” [28]. Therefore, as the usual pretest probability scores (Wells, Geneva) cannot be used with the same efficacy in cancer patients, the process of identifying biomarkers that could hold predictive diagnostic value and/or refine the existing scores is still ongoing.

An interesting study on the issue of thrombotic risk and NLR variation according to cancer type and disease staging was conducted by Howard et al. and published in 2019 [29]. The authors evaluated 5363 patients treated with first-line therapy for eight types of cancer (breast, pancreas, liver, esophagus, colon/rectum, prostate, ovary, and skin (melanoma)). The patients were stratified according to their demographic and clinical characteristics. Analysis performed for baseline NLR differences identified significantly higher baseline NLR for age ≥ 60, male gender, white race, and stage IV patients. The lowest pretreatment median NLR was observed in breast and prostate cancer patients, while the highest baseline NLR was observed in ovarian cancer patients. Although a high pretreatment NLR was associated with worse survival, the reported results of the strength association varied. After performance classification for the patient cohort as a whole and sensitivity/specificity analysis of the NLR cutoff as a predictor of overall patient survival, an optimal cutoff of 3.22 was proposed [29].

As the baseline NLR is modified in cancer and in a wide range of other inflammatory conditions, with NLR values reaching as high as ≥23 in terminal cancer patients [14], establishing NLR cutoff values for VTE risk assessment may be challenging. Data from individual studies show marked heterogeneity [30,31,32,33,34]. When it comes to NLR, according to a 2024 systematic review (113 studies) and meta-analysis (50 studies), a pre-chemotherapy NLR of ≥ 3 may hold predictive value for VTE in cancer patients [30]. This NLR ≥ 3 cutoff has also been proposed by other individual studies [31,32,33,34] (Table 3).

Further investigations of proposed cutoff values for NLR, PLR, and SII with positive diagnostic predictive value in non-cancer patients are summarized in Table 4.

NLR [36,37,40], PLR [36,37], and SII [37,38] have been associated with venous thromboembolic events, but the reported cutoffs vary throughout the available literature. A 2023 meta-analysis [36] tried to reconcile the controversies regarding NLR and PLR cutoffs with diagnostic value. Throughout 11 VTE studies, regarding NLR, a proposed NLR of >3 emerged (2.16 positive likelihood ratio and 0.4 negative likelihood ratio). In turn, when the diagnostic value of PLR was analyzed in seven VTE studies, a cutoff at PLR > 180 was identified (2.89 positive likelihood ratio and 0.45 negative likelihood ratio) [36]. Regarding the thrombus burden in deep vein thrombosis, Kuplay et al. [39] reported an association of NLR with both thrombus localization (NLR > 2.56 for proximal localization) and the number of venous segments involved (NLR values increasing with the number of venous segments affected), while PLR was correlated only with thrombus localization [39]. As mentioned before, SII is associated with acute VTE, with proposed cutoffs of SII > 755.54 for deep vein thrombosis [37] and SII > 1161 for predicting massive acute pulmonary embolism [38]. SII holds independent predictive value for massive acute PE, together with C-reactive protein and cardiac troponin, and SII values increase gradually with PE severity [38].

#### 2.2.2. Biomarkers with Proposed Predictive Value for Morbidity and Mortality (Short-Term and Long-Term) in Venous Thromboembolism

In addition to the predictive diagnostic value for acute venous thromboembolic events, complete blood count-derived immuno-inflammatory indices may refine the assessment of the risk of complications.

As the prevention of VTE-related morbidity depends on early diagnosis and treatment of thrombotic events, correct assessment of the thrombus burden in deep vein thrombosis allows for proactive prophylaxis of potential complications. A report by DeMartino et al. [41], following up on patients after iliofemoral deep vein thrombosis for 3–6 months, identified a cutoff value of NLR > 7.71 as a statistically significant positive predictor of evolution towards post-thrombotic syndrome when assessed together with other clinical markers at the time of DVT diagnosis [41] (Table 5).

NLR [42,43,44,45,46,47,48,50,51], PLR [42,43,44,46,47,48,49,50,51], and SII [42,43] are associated with mortality in acute pulmonary embolism, although the reported cutoffs vary (Table 5). Telo et al. [47] observed higher NLR and PLR levels in high-risk acute PE patients compared to low-risk patients (previously assessed with the sPESI score). These findings could be the mathematical consequence of higher neutrophil and platelet counts, together with the decreased lymphocyte counts that were observed by the investigators in the study group [47]. Another study by Karatas et al. [51], evaluating short-term (30-day) and long-term mortality in acute pulmonary embolism patients, observed significantly higher NLR and PLR levels at hospital admission in patients who died compared to those who survived, with Cox regression analysis demonstrating an independent correlation between total mortality and PESI scores, as well as elevated NLR and PLR [51]. Similarly, Ma et al. [50] reported the independent predictive value of NLR for mortality, with a 13% increase in 30-day mortality for every one unit of increase in NLR [50]. Another study by Duman et al. [44] reported NLR > 6 to be associated with an almost 13-fold increase in short-term (30-day) mortality, while NLR > 3.15 and age were independent risk factors for long-term (1-year) mortality [44].

Adverse outcomes after a venous thromboembolic event refer not only to mortality but also to other complications, such as major bleeding (Table 5). According to Siddiqui et al. [43], NLR, PLR, and SII were associated with a 90-day major bleeding risk after an acute VTE, with an NLR cutoff of 4.41 after both univariate and multivariate analyses [43].

CBC-derived indices can also be used for pulmonary embolism severity and risk stratification alongside the already established scores sPESI and PESI (Table 5). According to Telo et al. [47], NLR ≥ 3.56 and PLR ≥ 156 predict high sPESI [47]. Integrative models that improve the accuracy of sPESI by adding NLR have been proposed (Table 5), along with new scoring models such as CLOT-5 and the Naples prognostic score (NPS) (Table 6).

For instance, Siddiqui et al. [42] showed that patients with sPESI = 0 points and NLR ≤ 7.0 displayed superior sensitivity and negative predictive value compared to sPESI alone for 30-day mortality, suggesting the utility of NLR as a prognostic marker for short-term mortality in patients with very-low-risk PE [42]. Phan et al. [46] proposed adding 1 point each to the sPESI score for NLR > 5.46 or PLR > 256.6, demonstrating increased predictive value for all-cause mortality compared to the sPESI score alone [46] (Table 5). Similarly, the integration of NLR > 5.46 in the CLOT-5 score when assessing short-term (30-day) mortality risk showed the superiority of CLOT-5 compared to traditional predictive scores such as sPESI and PESI [52] (Table 7).

Another prognostic score that can be used to assess 30-day all-cause mortality in acute PE is the Naples prognostic score (Table 6). According to Zhu et al. [54], the rationale behind the choice of parameters included in the Naples prognostic score refers to their association with the development and progression of thrombosis. Hypoalbuminemia stimulates the liver to synthesize proteins (e.g., albumin, coagulation factors), resulting in a hypercoagulable status while promoting interstitial edema that leads to increased blood viscosity. Lipids intervene in the metabolism of the normal lung tissue, and the increased inflammatory response in acute pulmonary embolism can affect both cholesterol synthesis and hepatic absorption/transport of lipids, resulting in what has been described as the “lipid paradox” (lipid levels correlating negatively with mortality rates). The NLR and lymphocyte-to-monocyte ratio may be used as prognostic inflammatory markers, with LMR even being proposed as a surrogate marker of endothelial dysfunction [54]. The Naples prognostic score was found to be an independent risk factor for both 30-day all-cause mortality [54] and long-term mortality [53] in acute pulmonary embolism, non-inferior to PESI [53,54], and patients with NPS = 3–4 points had the highest risk of all-cause mortality compared to NPS = 1–2 or 0 points [54].

## 3. Discussion

The interplay between neutrophils and platelets is mathematically best reflected by the systemic immune-inflammation index as a biomarker derived from the complete blood count analysis. However, most of the literature published thus far focuses mainly on the NLR and PLR, with data on the SII being rather scarce by comparison. As demonstrated by a 2022 systematic review and meta-analysis by Ye et al. [55], higher SII is associated with an increased risk of cardiovascular disease (e.g., stroke, myocardial infarction, peripheral artery disease), with a similar trend for venous thrombosis, even though—according to the authors—the association for venous thrombosis was not statistically significant (hazard ratio = 4.65, 95%CI = 0.66–32.71, *p* = 0.122) [55]. As the topics of immunothrombosis and thromboinflammation have been increasing in popularity in the scientific community in recent years, with neutrophil–platelet interactions sparking more interest, further studies on SII reference values, as well as proposed cutoffs for various pathologies, are to be expected.

Thromboprophylaxis is another topic of interest to all fields of clinical medicine, including both medical and surgical specialties. Studies published in recent years propose various risk scores, and several recent reviews [19,20] have attempted to systemize the current knowledge and assess the validity of scoring systems for different populations. The implementation of standardized VTE risk assessment models (RAMs) has been recommended by official authorities such as the National Health Service (NHS) in England and the Center for Medicare and Medicaid Services in the United States when assessing thromboprophylaxis for inpatients [19]. However, the currently used validated scores do not include complete blood count-derived indices, and any newly developed scoring system would require external validation before implementation in clinical practice. Therefore, the ongoing research, although promising, requires refining.

A policy statement from the American Heart Association (AHA), formulated in a call for action regarding the prevention of VTE in hospitalized patients [56], was published in 2020. The document not only reinforced the importance of VTE risk assessment in all hospitalized patients, it also emphasized the need to report these data in a centralized manner, at a national level, in an effort to decrease venous thromboembolic events in hospitalized patients by 20% by 2030 [56]. Large datasets are required in order to further refine and update existing scoring systems, as well as to develop new ones; they would also facilitate a more accurate determination of biomarkers’ cutoffs, especially in cases of biomarkers with increased sensitivity and low specificity. An example is D-dimer, which is currently used in clinical practice for its negative predictive value, while for positive values, its quantitative measurement can, at most, be integrated into predictive scores (e.g., the IMPROVE-DD score).

There is also the question of actual usability. Personalized case management needs to consider multiple variables. Providing these data adds supplementary costs (e.g., from hiring the additional entry data personnel) and risks becoming time-consuming [19]. To avoid incomplete data collection, computer alert interventions [19] that prompt the user could be implemented.

Next, machine learning [19,57] could help refine the existing algorithms. Although useful for data classification, pattern recognition, and data optimization, machine learning algorithms in medicine have sparked a rather “controversial” and “heated discussion” because of ethical concerns [57]. Although machine learning models are being developed and proposed in various studies, the current challenges involve implementation gaps, as well as the need for external validation and regulatory approval [57]. A recent paper by Teodoru et al. [58], published in 2024, successfully implemented machine learning algorithms to integrate elevated NLR into pulmonary embolism risk stratification, with the help of cluster analysis, along with the classification and regression trees (CART) method, in a cohort of 160 patients with pulmonary embolism. The NLR cutoffs were defined based on the group’s median, and subgroup analysis of patients with a CBC determination available in the first 24 h from hospital admission showed slightly higher NLR values (4.69) than the entire group’s median NLR of 3.7. The proposed two-step cluster analysis proved to be a robust model when categorizing patients based on NLR, sPESI classification, and COVID-19 presence, while the CART algorithm further refined the findings, with high performance in predicting survival (97.3%), along with a 53.8% predictive ability for in-hospital mortality [58]. Clinical implementation of similar complex risk assessment models in general practice mandates more user-friendly interfaces (between the algorithms that organize and compute the electronic data and the physician who decides the individualized case management based on clinical reasoning and algorithm model results).

Last but not least, there is the issue of data availability. Complete blood count analysis has the advantages of being inexpensive and easily available in clinical practice. CBC-derived indices reflect the interactions between the immune system and hemostasis-preserving mechanisms. In venous thromboembolism, the NLR, PLR, and SII hold predictive value and complement severity assessment tools. However, as already presented in this review, data from individual studies are characterized by marked heterogeneity, reflected in various proposed cutoffs. Standardized future study designs, based on large datasets from national registries, could allow for the development of personalized cutoffs according to the patient’s profile, which could be further included in scoring systems.

## 4. Future Directions

Recommended future directions in research may include further exploring complete blood count-derived biomarkers in both healthy individuals and patients with established pathologies so as to increase their clinical applicability. Awareness programs dedicated to clinicians and patients on the topic of thromboembolic burden would further support data collection in national registries. Large datasets would allow for the identification of refined cutoffs according to the patient’s profile based on individual risk factors and comorbidities. All of these directions would support the comprehensive management of venous thromboembolism as part of a personalized medicine approach.

## 5. Conclusions

Neutrophils and platelets are key drivers of immunothrombosis and thromboinflammation, and their interplay is reflected in the dynamics of complete blood count-derived biomarkers.

The neutrophil-to-lymphocyte ratio (NLR), platelet-to-lymphocyte ratio (PLR), and systemic immune-inflammation index (SII) are associated with venous thromboembolic events (deep vein thrombosis and pulmonary embolism cases) in terms of both morbidity and mortality (short-term and long-term). However, the reported values are highly heterogeneous, and a consensus on cutoffs has not yet been established. Integrative models that add CBC-derived indices to already existing prediction scores, along with newly proposed scoring systems, have the potential to better reflect the complexity of thrombotic processes for a personalized approach to medicine.

## Figures and Tables

**Table 1:** Reported reference values for neutrophil-to-lymphocyte ratio (NLR), platelet-to-lymphocyte ratio (PLR), and systemic immune-inflammation index (SII).

Authors, Publication Year, Reference	Study Design	NLR Mean/Median Values	PLR Mean/Median Values	SII Mean/Median Values
Lee et al., 2018 [15]	*n* = 12,160 individuals without any medical history	1.65 (95%CI = 0.11–3.19)	132.40 (95%CI = 46.8–218)	Not determined
Fest et al., 2018 [16]	*n* = 8711 individuals aged 45 years and over	1.76 (95%CI = 0.83–3.92)	120 (95%CI = 61–239)	459 (95%CI = 189–1168)
Meng et al., 2017 [17]	*n* = 24,029 individuals aged 18–65 years	1.72 (IQR = 1.37–2.18)	108 (IQR = 89–132)	366 (IQR = 278–481)

Abbreviations: 95%CI—95% confidence interval; IQR—interquartile range.

**Table 2:** Risk assessment models for VTE risk in cancer patients.

Risk Assessment Models	Patient Population	Complete Blood Count Parameters Incorporated in Score Calculation
The Khorana risk score (KRS) [25]	- Non-surgical cancer outpatients initiating chemotherapy	Hemoglobin level < 100 g/L Pre-chemotherapy leucocyte count > 11 × 10^9^/L Pre-chemotherapy platelet count ≥ 350 × 10^9^/L
Vienna Cancer and Thrombosis Study (Vienna-CATS) nomogram score [26]	- Non-surgical cancer patients	
The Prospective Comparison of Methods for thromboembolic risk assessment with clinical Perceptions and AwareneSS in real-life patients with Cancer-Associated Thrombosis (COMPASS-CAT) score [27]	- Non-surgical cancer patients	Platelet count ≥ 350 × 10^9^/L

**Table 3:** Biomarkers with predictive value for VTE diagnosis in cancer patients.

Authors, Publication Year, Reference	Study Design	Investigated Biomarkers	Proposed Cutoffs	Other Results
Roy et al., 2024 [30]	- Systematic review (113 studies) and meta-analysis (50 studies) investigating the risk of VTE in cancer patients.	CBC parameters, factor VIII, time to peak thrombin, D-dimer, fibrinogen, NLR	- Hemoglobin < 100 g/L (measured at cancer diagnosis); - WBC > 11 × 10^9^/L (measured at cancer diagnosis); - Pre-chemotherapy NLR ≥ 3; - Preoperative platelet count ≥ 400 × 10^9^/L.	
Lekovic et al., 2023 [31]	- *n* = 816 polycythemia vera patients assessed for overall survival.	NLR, PLR	Upon univariate analysis, shorter overall survival for: Leukocyte count ≥ 15 × 10^9^/L (*p* < 0.001), Absolute neutrophil count ≥ 10 × 10^9^/L (*p* < 0.001), Platelet count ≥ 1000 × 10^9^/L (*p* = 0.027). Upon multivariate Cox regression model, survival predictor: Absolute neutrophil count ≥ 10 × 10^9^/L (HR = 1.6; *p* = 0.001).	- NLR and PLR were predictors of arterial thrombosis development but not of venous thrombosis; - Other factors associated with shorter overall survival: previous thrombosis (*p* = 0.03), development of arterial (*p* < 0.001) and venous (*p* < 0.001) thrombosis during follow-up (univariate analysis).
Otasevic et al., 2022 [32]	- Prospective study; - *n* = 706 lymphoma patients followed for VTE events over a median of 25 months.	NLR, PLR, CRP, ESR, LDH, total protein, albumin	- NLR ≥ 3 (65.2% sensitivity, 57.1% specificity); - PLR ≥ 10 (69.6% sensitivity, 49.7% specificity); - CRP > 20 mg/L (71.7% sensitivity, 63.7% specificity).	- Significantly higher values of NLR, PLR, CRP, ESR, and LDH in the VTE group; - Significantly lower total protein and albumin in the VTE group; - Prognostic value of NLR, PLR, total protein, albumin, LDH, and CRP for VTE development (univariate regression analysis); - Independent prognostic value of NLR and CRP for VTE development (multivariate regression analysis).
Grilz et al., 2018 [33]	- Prospective study; - *n* = 1469 newly diagnosed or progressing solid cancer patients, followed up for thrombotic events (venous or arterial) for 2 years.	NLR, PLR		- No statistically significant association between NLR and VTE occurrence in patients with cancer; - Increased NLR and PLR independently associated with increased risk of mortality.
Ferroni et al., 2015 [34]	- *n* = 810 primary or relapsing solid cancer outpatients, at the start of a new chemotherapy regimen, followed up for a first VTE event during chemotherapy for a median of 9 months; - Risk category assessment was performed using the Khorana clinical model [25] for predicting chemotherapy-associated VTE.	NLR, PLR	- NLR > 3 (~59% sensitivity, 57% specificity, negative predictive value 95%, AUC = 0.55, SE = 0.04); - PLR > 260 (~30% sensitivity, 80% specificity, negative predictive value 94%, AUC = 0.54, SE = 0.04).	- 6.7% VTE occurrence rate, but 47% incidental VTE diagnosed at the time of restaging; - Significantly higher median pre-chemotherapy NLR and PLR in patients with intermediate-risk class who developed symptomatic VTE; - NLR > 3 or PLR > 260 held ~2-fold risk of VTE events; - Independent predictive value of PLR in intermediate-risk cancer patients (multivariate models of Cox proportional hazards survival analysis); - NLR associated with the occurrence of arterial thrombosis, but not with VTE.

Abbreviations: AUC—area under the curve; CBC—complete blood count; CRP—C-reactive protein; ESR—erythrocyte sedimentation rate; HR—hazard ratio; LDH—lactate dehydrogenase; NLR—neutrophil-to-lymphocyte ratio; PLR—platelet-to-lymphocyte ratio; SE—standard error; VTE—venous thromboembolism; WBC—white blood cell.

**Table 4:** Biomarkers with predictive value for VTE diagnosis in non-cancer patients.

Authors, Publication Year, Reference	Study Design	Investigated Biomarkers	Proposed Cutoffs	Other Results
Nguyen et al., 2024 [35]	- Retrospective study; - *n* = 585 patients suspected of thrombosis (281 newly diagnosed VTE, 82 newly diagnosed arterial thrombosis, and 222 patients not confirmed to have thrombosis).	CBC parameters, fibrinogen, D-dimer		- NLR associated with the occurrence of arterial thrombosis, but not with VTE.
Hu et al., 2023 [36]	- Meta-analysis of 11 VTE studies.	NLR, PLR	- NLR > 3 (76% pooled sensitivity, 71% pooled specificity, estimated DOR = 5.3 with SROC-AUC = 0.74, 95%CI = 0.70–0.78); - PLR > 180 (61% pooled sensitivity, 74% pooled specificity, estimated DOR = 6.64 with SROC-AUC = 0.79, 95%CI = 0.76–0.83).	- Moderate diagnostic accuracy of calculated NLR and PLR for patients with DVT.
Tort et al., 2023 [37]	- Retrospective study; - *n* = 126 patients (*n* = 63 DVT patients and *n* = 63 healthy controls) included in the study after propensity matching (to eliminate differences between groups) from the initial population of *n* = 155 DVT patients versus *n* = 179 healthy controls.	CBC parameters, NLR, PLR, SII	- NLR >3 (~71% sensitivity, ~69% specificity, AUC = 0.8, 95%CI = 0.75–0.85, *p* < 0.001); - PLR > 142.66 (~70% sensitivity, ~69% specificity, AUC = 0.79, 95%CI = 0.74–0.84, *p* = 0.01); - SII > 755.54 (~78% sensitivity, ~73% specificity, AUC = 0.86, 95%CI = 0.82–0.9, *p* < 0.001);	- Significantly lower hemoglobin, hematocrit, and lymphocyte count in the DVT group; - Significantly higher WBC, neutrophil count, platelet count, NLR, PLR, SII, and mean platelet volume in the DVT group.
Gok et al., 2021 [38]	- Retrospective study; - *n* = 442 patients with acute PE.	SII, cardiac troponin, CRP, D-dimer, NT-proBNP	- SII > 1161 (91% sensitivity, 90% specificity, AUC = 0.96, 95%CI = 0.94–0.98, *p* < 0.001) for predicting massive acute PE.	- Significantly higher WBC, neutrophil count, platelet count, cardiac troponin, SII, D-dimer, and NT-proBNP levels in patients with massive acute PE; - Significantly lower lymphocyte count in patients with massive acute PE; - Significantly higher SII in massive acute PE versus submassive and non-massive PE, with SII values increasing gradually with PE severity (*p* < 0.001); - Higher SII levels in patients who suffered in-hospital death after PE. - Independent predictive value of SII for massive acute PE (OR = 1.005, 95%CI = 1.002–1.007, *p* < 0.001), together with C-reactive protein and cardiac troponin.
Kuplay et al., 2020 [39]	- Retrospective study; - *n* = 933 patients with confirmed DVT, assessed for DVT burden based on thrombus localization and number of vein segments involved.	NLR, PLR	- NLR > 2.56 for proximal DVT (65.4% sensitivity, 55.0% specificity, AUC = 0.6, 95%CI = 0.56–0.64, *p* < 0.001).	- Mean NLR values were higher in proximal DVT (femoral and iliac veins) compared to distal DVT (crural and popliteal veins), *p* = 0.05; - Mean PLR values were higher for iliac localization compared to femoral, crural, and popliteal DVT (*p* = 0.03); - NLR augmented with the number of vein segments involved (*p* = 0.001); - PLR did not correlate with the number of vein segments affected.
Farah et al., 2019 [40]	- *n* = 327 patients with an initial diagnosis of acute VTE, further classified as *n* = 272 confirmed VTE versus *n* = 55 patients without VTE as the control group.	NLR, PLR, MPV	- NLR > 5.3 (69% sensitivity, 57% specificity, AUC = 0.67, 95%CI = 0.60–0.75, *p* < 0.001); - MPV > 8.6 (52% sensitivity, 67% specificity, AUC = 0.61, 95%CI = 0.53–0.68, *p* = 0.014).	- Significantly higher NLR (*p* < 0.001), MPV (*p* = 0.008), and PLR (*p* = 0.014) in the acute VTE group compared to controls; - NLR (OR 1.2, 95%CI = 1.01–1.4, *p* = 0.041) and MPV (OR 1.5, 95%CI = 1.07–2.12, *p* = 0.5) were associated with acute VTE (multivariate logistic regression model).

Abbreviations: AUC—area under the curve; CBC—complete blood count; CI—confidence interval; CRP—C-reactive protein; DOR—diagnostic odds ratio; MPV—mean platelet volume; NLR—neutrophil-to-lymphocyte ratio; NT-proBNP—N-terminal pro-B-type natriuretic peptide; OR—odds ratio; PLR—platelet-to-lymphocyte ratio; SII—systemic immune-inflammation index; SROC—summary receiver operating characteristic curve; WBC—white blood cell.

**Table 5:** Biomarkers with predictive value for VTE morbidity and mortality.

Authors, Publication Year, Reference	Study Design	Investigated Biomarkers	Proposed Cutoffs	Other Results
DeMartino et al., 2024 [41]	- Retrospective study; - *n* = 118 patients with iliofemoral DVT, followed up for 3–6 months for the occurrence of a post-thrombotic syndrome.	NLR	NLR > 7.71 at the time of iliofemoral DVT: predictive value for evolution towards post-thrombotic syndrome (AUC = 0.63)	- NLR is a statistically significant positive predictor, when assessed together with other clinical markers at the time of DVT diagnosis (OR = 1.83, 95%CI = 1.20–2.78; *p* = 0.005).
Siddiqui et al., 2024 [42]	- Retrospective study; - Data of patients with acute PE from the Computerized Registry of Patients with Venous Thromboembolism (RIETE) (*n* = 10085 patients) and Loyola University Medical Center (LUMC) (*n* = 700 patients), assessed for the prognostic value of NLR, PLR, and SII compared to sPESI alone, in terms of 30-day all-cause mortality as the primary outcome.	NLR, PLR, SII	NLR > 7 (aOR = 3.46; 95%CI = 2.60–4.60) PLR > 220 (aOR = 2.36; 95%CI = 1.77–3.13) SII > 1600 (aOR = 2.52; 95%CI = 1.90–3.33)	- NLR, PLR, and SII were associated with mortality in acute PE, the association being strongest for NLR compared to PLR and SII (upon multivariate analysis); - Patients with sPESI = 0 points and NLR ≤ 7.0 displayed superior sensitivity and negative predictive value compared to sPESI alone for 30-day mortality, suggesting the utility of NLR as a prognostic marker for 30-day mortality in very-low-risk PE.
Siddiqui et al., 2022 [43]	- Retrospective study; - Assessment of 90-day adverse outcomes after an acute VTE, using data of *n* = 4487 patients from the Computerized Registry of Patients with Venous Thromboembolism (RIETE) database: *n* = 2683 symptomatic PE, 283 incidental PE, 1129 DVT, 175 upper-limb DVT, 69 splanchnic vein thrombosis, 142 superficial vein thrombosis, and 20 retinal vein thrombosis.	NLR, PLR, SII	Upon univariate analysis: NLR > 4.41: risk of major bleeding NLR > 4.96: risk of death PLR > 166.47: risk of major bleeding PLR > 167.96: risk of death SII > 1154.81: risk of major bleeding SII > 1134.50: risk of death Upon multivariate analysis: NLR > 4.41: risk for major bleeding (aOR = 1.73; 95%CI = 1.05–2.86) NLR > 4.96: risk of death (aOR = 2.50, 95%CI = 1.83–3.42) SII > 1134.5: risk of death (aOR: 1.52, 95%CI = 1.08–2.14)	Upon univariate analysis: - The overall accuracy for major bleeding events: NLR: 0.67 (95%CI = 0.62–0.72); PLR: 0.62 (95%CI = 0.58–0.67); SII: 0.66 (95%CI = 0.61–0.71). - The overall accuracy for mortality: NLR: 0.73 (95%CI = 0.71–0.76); PLR: 0.63 (95%CI = 0.60–0.66); SII: 0.7 (95%CI = 0.67–0.77). Upon multivariate analysis: PLR > 167.96 or PLR > 166.47: no increased risk for major bleeding and mortality. SII >1134.5: no increased risk for major bleeding.
Duman et al., 2021 [44]	- Retrospective study; - *n* = 828 patients with acute PE, assessed for short-term (30-day) and long-term (1-year) mortality.	NLR, PLR, PLT/MPV, CRP	NLR > 6.1 (75% sensitivity, 75.6% specificity, AUC = 0.75, *p* = 0.017) for short-term mortality NLR > 3.1 (68.6% sensitivity, 59.8% specificity, AUC = 0.67, *p* < 0.001) for predicting 1-year mortality PLR > 152.3 (64% sensitivity, 52% specificity, AUC = 0.64, *p* < 0.001) for predicting 1-year mortality	- NLR and CRP were significantly higher in the mortality group for both short-term and long-term mortality; - PLR was significantly higher in non-survivors at the 1-year follow-up; - PLT/MPV showed no significant difference between survivors and non-survivors in the 30-day and 1-year follow-up periods; - NLR > 6.1 was associated with a ~13-fold increase in short-term mortality; - NLR > 3.25 was associated with a ~3-fold increase in mortality over 180 days, while NLR > 3.14 was associated with a 2.2-fold increase in 1-year mortality; - NLR > 3.15 and age were independent risk factors for long-term mortality.
Efros et al., 2021 [45]	- Retrospective study; - *n* = 2072 patients with acute PE, assessed for short-term (30-day) and long-term mortality.	NLR	Higher 30-day mortality risk for NLR > 5.12 (aOR = 2.82; 95%CI = 2.14–3.70, *p* < 0.001) Higher 1-year mortality for NLR > 5.12 (aOR = 2.51, 95%CI = 2.04–3.08, *p* < 0.001).	- NLR in acute PE was associated with in-hospital mortality, a longer duration of hospitalization, and a worse short-term and long-term prognosis.
Phan et al., 2020 [46]	- Retrospective study; - *n* = 191 patients with acute PE, assessed for all-cause mortality.	NLR, PLR, BNP, lactate, troponin	NLR > 5.46 (75% sensitivity, 66.9% specificity, AUC = 0.69, 95%CI = 0.57–0.82, *p* <0.01) PLR > 256.6 (53.6% sensitivity, 82.2% specificity, AUC = 0.69, 95%CI = 0.58–0.81, *p* < 0.01)	- Significant difference in PLR (*p* = 0.02), but not NLR, between patients with low-risk, submassive, and massive-risk PE; - High NLR and PLR were associated with all-cause mortality (*p* < 0.01); - When predicting all-cause mortality, an integrated model, adding 1 point each to the sPESI score for NLR > 5.46 or PLR > 256.6, demonstrated increased predictive value (AUC = 0.78, 95%CI = 0.68–0.87, *p* < 0.01) compared to the sPESI score alone (AUC = 0.73, 95%CI = 0.63–0.83, *p* < 0.01).
Telo et al., 2019 [47]	- Retrospective study; - *n* = 82 patients with acute PE, stratified for mortality risk at 30 and 90 days.	D-dimer, troponin I, BNP, NLR, PLR	NLR ≥ 3.56 for predicting high sPESI (66% sensitivity, 53% specificity, AUC = 0.68, 95%CI = 0.56–0.79, *p* < 0.05) PLR ≥ 156 for predicting high sPESI (74% sensitivity, 64% specificity, AUC = 0.70, 95%CI = 0.59–0.82, *p* < 0.01) NLR ≥ 4.82 for predicting total mortality (AUC = 0.718, 95%CI = 0.51–0.93, *p* < 0.05) PLR ≥ 229.66 for predicting total mortality (AUC = 0.72, 95%CI = 0.54–0.9, *p* < 0.05)	- Statistically significant higher mean BNP (*p* < 0.01), TnI (*p* < 0.05), neutrophil count (*p* < 0.05), platelet count (*p* < 0.05), NLR (*p* < 0.01), and PLR (*p* < 0.01) in high-risk compared to low-risk patients (sPESI); - Statistically significant lower lymphocyte count (*p* < 0.01) in high-risk compared to low-risk patients (sPESI); - When comparing the high-risk versus low-risk patients (sPESI), increased total 3-month mortality observed for PLR > 156, along with increased hospital mortality and total 3-month mortality for NLR > 3.56.
Kasapoğlu et al., 2019 [48]	- Retrospective study; - *n* = 550 patients with acute PE, assessed for all-cause short-term (30-day) mortality using sPESI.	NLR, PLR, D-dimer, NT-proBNP	NLR > 7.3 (69.7% sensitivity, 47.5% specificity, AUC = 0.604, 95%CI = 0.53–0.68, *p* = 0.003) PLR > 170 (63% sensitivity, 53% specificity, AUC = 0.582, 95%CI = 0.5–0.66, *p* = 0.022) D-dimer > 1.6 μg/mL (66% sensitivity, 58% specificity, AUC = 0.627, 95%CI = 0.51–0.74, *p* = 0.036) NT-proBNP > 1300 pg/mL (71% sensitivity, 54% specificity, AUC = 0.710, 95%CI = 0.63–0.79, *p* < 0.001) sPESI > 2 (84% sensitivity, 89% specificity, AUC = 0.895, 95%CI = 0.86–0.93, *p* < 0.001)	- Significantly higher baseline NLR, PLR, NT-proBNP, and D-dimer in patients who died within 30 days; - NLR showed a weak positive correlation with sPESI score (r = 0.100, *p* = 0.01) and NT-proBNP (r= 0.232, *p* < 0.001); - Significant independent prognostic factors of short-term mortality in APE: only patient risk status (HR = 2.83, 95%CI = 1.96–8.35, *p*= 0.038) and sPESI score (HR = 28.33, 95%CI = 5.88–55.36, *p* < 0.001) (multivariate Cox regression); - After subgroup analysis, in patients without comorbid diseases, predictors of mortality in APE were the NLR (*p* = 0.016), patient’s risk status (*p* = 0.026), and sPESI score (*p* < 0.001).
Cetin et al., 2017 [49]	*n* = 459 patients with acute PE, assessed for in-hospital and long-term adverse outcomes, and followed up for all-cause mortality for a median of 28 months.	PLR, cTnI		- Patients with PLR in the highest tertile had more frequent in-hospital adverse events and long-term all-cause mortality; - PLR was a significant predictor of both in-hospital adverse events (OR = 1.59, 95%CI = 1.12–2.15, *p* = 0.004) and long-term all-cause mortality (OR = 1.75, 95%CI = 1.21–2.87, *p* = 0.001); - PLR was significantly correlated with RV/LV ratio and cTnI levels.
Ma et al., 2016 [50]	*n* = 248 patients with acute PE, assessed for 30-day mortality.	NLR, PLR	NLR > 5.99 (80% sensitivity, 66.7% specificity, AUC = 0.79, 95%CI = 0.70–0.88, *p* < 0.001) PLR > 325 (65% sensitivity, 80.7% specificity, AUC = 0.79, 95%CI = 0.7–0.88, *p* < 0.001)	NLR has independent predictive value for mortality, with a 13% increase in 30-day mortality for every 1 unit of increase in NLR (OR = 1.13, 95%CI = 1.04–1.23).
Karatas et al., 2016 [51]	- Retrospective study; - *n* = 203 patients with PE, assessed for short-term (30-day) and long-term mortality, followed up for a median of 20 months.	NLR, PLR	Predictors of total mortality: NLR > 5.93 (87.8% sensitivity, 74.5% specificity, AUC = 0.84, *p* = 0.01); PLR > 191 (60.6% sensitivity, 83.2% specificity, AUC = 0.73, *p* = 0.01).	- Significantly higher NLR and PLR levels at hospital admission in patients who died (regardless of death occurring within short-term or long-term follow-up); - Independent correlation between total mortality and PESI scores (HR = 1.02, 95%CI = 1.01–1.04, *p* = 0.01), respectively elevated levels of NLR (HR = 1.13, 95%CI = 1.04–1.23, *p* = 0.01) and PLR (HR = 1.002, 95%CI = 1.001–1.004, *p* = 0.01) (Cox regression analysis).

Abbreviations: aOR—adjusted odds ratio; AUC—area under the curve; BNP—brain natriuretic peptide; 95%CI—95% confidence interval; CRP—C-reactive protein; cTnI—cardiac troponin I; HR—hazard ratio; MPV—mean platelet volume; NLR—neutrophil-to-lymphocyte ratio; NT-proBNP—N-terminal pro-B-type natriuretic peptide; OR—odds ratio; PESI—Pulmonary Embolism Severity Index; PLR—platelet-to-lymphocyte ratio; ROC—receiver operating characteristic curve; SII—systemic immune-inflammation index; sPESI—simplified PESI score; TnI—troponin I.

**Table 6:** Proposed prediction scores for mortality in acute pulmonary embolism.

CLOT-5 Prediction Score for 30-Day Mortality in Acute PE	Naples Prognostic Score (NPS) for 30-Day All-Cause Mortality in Acute PE
Cancer	Albumin < 4 g/dL (1 point)
Lactic acidosis (lactic acid > 2 mm/L)	Total cholesterol ≤ 180 mg/dL (1 point)
Oxygen saturation < 90%	NLR > 2.96 (1 point)
Tachycardia > 120 bpm	LMR ≤ 4.44 (1 point)
+ five other variables:	
Requirement of at least one pressor NLR > 5.46 RDW > 15% RV/LV ratio > 1.4 (marker of RV strain) Right ventricular outflow tract velocity time integral < 9.5 cm (marker of decreased RV stroke volume)	NPS maximum score: 4 points

Abbreviations: LMR—lymphocyte-to-monocyte ratio; LV—left ventricle; NLR—neutrophil-to-lymphocyte ratio; NPS—Naples prognostic score; RDW—red cell distribution width; RV—right ventricle.

**Table 7:** Biomarkers included in scoring systems for pulmonary embolism mortality.

Authors, Publication Year	Study Design	Investigated Biomarkers	Proposed Cutoffs	Other Results
Marginean et al., 2024 [52]	- Retrospective study; - *n* = 488 confirmed acute PE, assessed with CLOT-5 score for 30-day mortality.	Lactic acid, NLR, RDW	NLR > 5.46 RDW > 15%	- Superiority of CLOT-5 score compared to traditional predictive scores such as sPESI and PESI: AUC = 0.90 ± 0.29 (CLOT-5) compared to AUC = 0.73 ± 0.55 (sPESI) and AUC = 0.79 ± 0.43 (PESI).
Pay et al., 2024 [53]	- *n* = 239 patients with acute PE, followed up for a mean of 24 months; - Patients were categorized based on the Naples prognostic score for long-term mortality assessment.	Albumin, total cholesterol, LMR, NLR	NLR > 2.96 LMR ≤ 4.44	- Naples prognostic score (HR = 1.65, 95%CI = 1.22–2.24, *p* = 0.001) and sPESI (HR = 1.89; 95%CI = 1.22–2.91; *p* = 0.004) were independent predictors for long-term mortality, with NPS being non-inferior to sPESI.
Zhu et al., 2023 [54]	- Retrospective study; - *n* = 325 patients hospitalized with acute PE, assessed for short-term (30-day) all-cause mortality; - Patients were categorized based on the Naples prognostic score (*n* = 131 with NPS = 0, *n* = 153 with NPS = 1–2, *n* = 41 with NPS = 3–4) and based on short-term mortality (*n* = 294 survivors, *n* = 31 non-survivors).	CBC, total cholesterol, albumin, D-dimer, troponin, NT-proBNP, arterial blood gas analysis markers (pH, lactate, partial pressure of oxygen [PaO_2_], and bicarbonate)	NLR > 2.96 LMR ≤ 4.44	- NPS predicts all-cause 30-day mortality (AUC = 0.78, 95%CI = 0.68–0.86, 80.6% sensitivity, 72.1% specificity); - NPS is an independent risk factor for 30-day all-cause mortality in acute PE (*p* = 0.0004) (Cox multivariate analysis); - When assessing ROC curves for NPS versus PESI, the AUC for the predictive value of NPS was not lower than that of PESI; - Compared to NPS = 0 points, the risk of all-cause 30-day mortality in patients with acute PE was significantly increased by 239% for NPS 1–2 points (HR = 3.385, *p* = 0.031) and by 338% for NPS 3–4 points (HR = 4.377, *p* = 0.023).

Abbreviations: AUC—area under the curve; 95%CI—95% confidence interval; HR—hazard ratio; LMR—lymphocyte-to-monocyte ratio; NPS—Naples prognostic score; NLR—neutrophil-to-lymphocyte ratio; PESI—Pulmonary Embolism Severity Index; RDW—red cells distribution width; ROC—receiver operating characteristic curve; sPESI—simplified PESI score.
